# Mutation of FMDV L^pro^ H138 residue drives viral attenuation in cell culture and *in vivo* in swine

**DOI:** 10.3389/fvets.2022.1028077

**Published:** 2022-10-31

**Authors:** Paul A. Azzinaro, Gisselle N. Medina, Devendra Rai, Elizabeth Ramirez-Medina, Edward Spinard, Monica Rodriguez-Calzada, James Zhu, Elizabeth Rieder, Teresa de los Santos, Fayna Díaz-San Segundo

**Affiliations:** ^1^Plum Island Animal Disease Center, Agricultural Research Service, U.S. Department of Agriculture, Greenport, NY, United States; ^2^National Bio and Agro-Defense Facility (NBAF), Agricultural Research Service (ARS), U.S. Department of Agriculture (USDA), Manhattan, KS, United States; ^3^Pfizer Worldwide Research, Development and Medical, Pearl River, NY, United States; ^4^Oak Ridge Institute for Science and Education, PIADC Research Participation Program, Oak Ridge, TN, United States

**Keywords:** FMDV, foot-and-mouth disease virus, leader proteinase, L^pro^, attenuated virus

## Abstract

The foot-and-mouth disease virus (FMDV) leader proteinase (L^pro^) is a papain like protease that cleaves the viral polyprotein and several host factors affecting host cell translation and induction of innate immunity. Introduction of L^pro^ mutations ablating catalytic activity is not tolerated by the virus, however, complete coding sequence deletion or introduction of targeted amino acid substitutions can render viable progeny. In proof-of-concept studies, we have previously identified and characterized FMDV L^pro^ mutants that are attenuated in cell culture and in animals, while retaining their capacity for inducing a strong adaptive immunity. By using molecular modeling, we have now identified a His residue (H138), that resides outside the substrate binding and catalytic domain, and is highly conserved across serotypes. Mutation of H138 renders possible FMDV variants of reduced virulence *in vitro* and *in vivo*. Kinetics studies showed that FMDV A12-L_H138L_ mutant replicates similarly to FMDV A12-wild type (WT) virus in cells that do not offer immune selective pressure, but attenuation is observed upon infection of primary or low passage porcine epithelial cells. Western blot analysis on protein extracts from these cells, revealed that while processing of translation initiation factor eIF-4G was slightly delayed, no degradation of innate sensors or effector molecules such as NF-κB or G3BP2 was observed, and higher levels of interferon (IFN) and IFN-stimulated genes (ISGs) were induced after infection with A12-L_H138L_ as compared to WT FMDV. Consistent with the results in porcine cells, inoculation of swine with this mutant resulted in a mild, or in some cases, no clinical disease but induction of a strong serological adaptive immune response. These results further support previous evidence that L^pro^ is a reliable target to derive numerous viable FMDV strains that alone or in combination could be exploited for the development of novel FMD vaccine platforms.

## Introduction

Foot-and-mouth disease virus (FMDV) is the prototype member of the *Aphthovirus* genus of the *Picornaviridae* family. The virus contains a positive single-stranded RNA genome of approximately 8,500 nucleotides surrounded by a non-enveloped icosahedral protein capsid. Upon infection, the viral RNA is released in the cytoplasm of the cell followed by rapid translation into a single polyprotein that is co-temporarily processed into intermediate and mature proteins, by three viral-encoded products, leader (L^pro^), 2A and 3C^pro^. Final viral cleavage products include four structural, VP1, VP2, VP3, and VP4, and 10 non-structural (NS) proteins (L^pro^, 2A, 2B, 2C, 3A, 3B_1, 2, 3_, 3C^pro^, and 3D^pol^) although, as mentioned above, several intermediate protein products are also detected during the viral infectious cycle (1). In particular, L^pro^ cleaves itself off from the rest of the viral polyprotein at its carboxyl terminus (2, 3). FMDV displays high genetic variation and exists as a quasipecies in seven distinct serotypes including A, O, C, Asia and South African Territories (SAT)−1, 2, and 3, and multiple strains. Such a genetic variability is probably the result of its error-prone RNA polymerase (3D^pol^), the multiple receptor usage, and the virus adaptation evolved to successfully infect over 70 cloven-hoof animal species (4, 5).

Outbreaks of FMD can cause devastating economic loses in FMD-free countries and, at the same time, they can jeopardize development of nations that rely on agriculture for subsistence (6). Control of FMD outbreaks is mainly achieved through a strong veterinary surveillance, physical isolation of endemic areas, and use of an inactivated whole virus vaccine in endemic/high risk areas as well as in some FMD-free areas to maintain the “free” status (7). Although this vaccine has proven successful in reducing the number of outbreaks worldwide, it has fallen short in eradicating FMD probably due to intrinsic limitations of the vaccine *per se*, resource crunch and social and political instability in endemic areas (8). There is interest in developing alternative vaccines that could be tailored for using in different environments (9). One of the main limitations of the current vaccine is the need of 7 days to induce protection. Historically, it has been shown that live attenuated vaccines (LAVs) can act rapidly post inoculation inducing strong immunity as early as 3 days post- immunization (10). In fact, viral disease eradication has only been achieved for smallpox and rinderpest by using LAV (11, 12). Thus far, no attenuated vaccine has been successfully developed for FMDV (9). However, previous studies showed that viable attenuated FMDV could be derived using reverse genetics by deleting the entire L^pro^ coding region (13). Unfortunately, cattle and swine studies showed limited replication of this virus, poor immune response and lack of protection in efficacy trials (14, 15). Nevertheless, leaderless strains (LLV) have been further developed by inclusion of markers that allow differentiation of infected from vaccinated animals (DIVA) and convenient restriction sites for easy genetic manipulation, leading to the development of an effective platform for production of inactivated FMDV vaccine (16). Moreover, the LLV strain has been of extreme utility in studies aimed to deciphering the mechanisms by which FMDV counteracts the immune response in the host. It is currently known that L^pro^ blocks the induction of interferon (IFN) mRNA and the expression of IFN protein (17–21). In addition, the molecular mechanisms implicated in this inhibition have been further elucidated, and to this end many signaling molecules have been identified as L^pro^ specific targets in a direct or indirect manner [reviewed in (22–25)].

We and others have previously shown that viable attenuated FMDV could also be derived after introduction of specific mutations in the conserved L^pro^ SAP domain (26–28) or in other residues that abolish specific L^pro^ enzymatic activities such as deUbiquitinase and deISGylase activity (22, 24, 29). Interestingly, and differently than for leaderless virus, inoculation with SAP mutant FMDV induces a strong adaptive immune response and animals are completely protected against challenge with wild type virus as early as 2 days post-vaccination (dpv) and for at least 21 dpv. Similarly, a W105A mutation that obliterates deUb- and deISG- activity, resulted in a virus attenuated in cell culture and in a mouse model of FMD (22).

In this work, we have identified H138 as another highly conserved amino acid within L^pro^ that lies outside of the substrate-binding pocket, but plays a critical role in FMDV virulence. While a conserved change such as H138N did not dramatically affect virus growth properties and characteristics, disruptive H138L did result in a virus with slower kinetics of growth in cell culture allowing for the induction of higher levels of IFN and IFN stimulated genes (ISGs). Interestingly, inoculation of swine with this FMDV A12-L_H138L_ mutant induced mild or no disease, albeit induction of significant and protective levels of neutralizing antibodies. These results highlight the plasticity of L^pro^ as a candidate target within the viral genome, to derive multiple virus strains with potential for development as novel live attenuated, or relatively safer seeds of inactivated vaccines, for the control of FMD.

## Materials and methods

### Cells and viruses

BHK-21 cells (baby hamster kidney cells strain 21, clone 13, ATCC CL10), obtained from the American Type Culture Collection (ATCC, Rockville, MD) were used to propagate virus stocks and to measure virus titers. Porcine kidney (LF-BK, SK6, and IBRS-2) cells were obtained from the Foreign Animal Disease Diagnostic Laboratory (FADDL), Animal, Plant, and Health Inspection Service (APHIS) at the PIADC and/or ATCC (Manassas, VA). Primary/secondary porcine kidney (PK) cells were provided by the APHIS National Veterinary Service Laboratory, Ames, Iowa. LF-BK, IBRS-2, SK6 and PK cells were maintained in minimal essential medium (MEM, Thermo Fisher, Waltham, MA) containing 10% fetal bovine serum (FBS) and supplemented with 1% antibiotics and non-essential amino acids. BHK-21 were maintained in similar media but bovine calf instead of fetal bovine serum was used, and the media was also supplemented with 10% Tryptose phosphate broth (Thermo Fisher). All cell cultures were incubated at 37°C in 5% CO_2_.

FMDV A12-WT was generated from the full-length serotype A12 infectious clone, pRMC35 (30). FMDV A12-LLV (leaderless virus) was derived from the infectious clone by deleting the Lb coding region, pRM-LLV2 (13). A12-L_H138_ mutant viruses were constructed by site directed mutagenesis using the QuickChange^®^ kit (Agilent, La Jolla, CA). All viruses were derived by electroporation of RNA in BHK-21 cells, passed 4 times in the same cells, followed by propagation, concentration by polyethylene glycol precipitation, and titration by plaque assay (pfu/ml) or end point dilution (TCID_50_/ml) on BHK-21 cells (30). Viral full-length sequences were confirmed by DNA sequencing of derived viral cDNA using an ABI prism 7,000 apparatus (Applied Biosystems, Thermo Fisher).

### Molecular modeling

Initial structural analysis was performed using the crystal structure of the Protein Data Bank (PDB): 4QBB (31). The model was manipulated using Discovery Studio visualizer (v21.1.0.20298). Structural analysis identified internal interacting residues which we predicted to effect protein stability. The identified critical residue was mutated *in silico* and the effects of substitutional mutations were simulated.

### Western blotting

Total cell lysates were prepared as described previously (20, 23). Proteins were resolved by SDS-PAGE and analyzed by western blotting. eIF4G was detected with a rabbit polyclonal antibody (Ab) #A300-502A (Bethyl Laboratories, Montgomery, TX), p65 with a rabbit polyclonal Ab #RB-1638 (NeoMarkers, Lab Vision, Freemont, CA), G3BP2 with a rabbit polyclonal Ab #OALA09398 (Aviva Systems Biology, San Diego, CA), VP1 with a rabbit polyclonal Ab made at PIADC and control tubulin-α, with mouse monoclonal Ab Ab-2 MS-581 (NeoMarkers, Lab Vision, Freemont, CA). Secondary Abs anti-mouse or anti-rabbit, conjugated to horseradish peroxidase (HRP) were obtained from Pierce (Rockford, IL). Protein bands were detected with ECL chemiluminescence Kit (Biorad, Hercules, CA) and images acquired with Gel Doc c300 digital imager (Azzure Biosystems, Dublin, CA).

### Detection of interferon (IFN) stimulated genes (ISGs) by real time PCR

Expression of several ISGs was analyzed by qRT-PCR as previously described (27). RNA was extracted from PK cells infected at a multiplicity of infection (MOI) of 10, with wild type (WT), leaderless (LLV) or mutant H138L FMDV. Porcine glyceraldehyde-3-phosphate dehydrogenase (GAPDH) was used as the internal control to normalize the values for each sample. Reactions were performed in an ABI Prism 7500 sequence detection system (Applied Biosystems). Relative mRNA levels were determined by comparative cycle threshold analysis (user bulletin 2; Applied Biosystems) utilizing as a reference the samples at 0 dpi.

### Analysis of IFN-α protein

A porcine IFN-α (pIFN-α) double capture ELISA previously developed in our laboratory was used to quantitate pIFN-α protein in the supernatants of infected cells (32). Anti pIFN-α mAb K9 and F17 were purchased from R&D Systems (Minneapolis, MN). MAb K9 (1 μg/ml) was used for antigen capture and biotinylated mAb F17 (0.35 μg/ml) in conjunction with horse-radish-peroxidase-conjugated streptavidin (streptavidin-HRP) (KPL, Gaithersburg, MD) were used for detection. pIFN-α concentrations were determined by extrapolation on a standard curve prepared with recombinant pIFN-α (PBL Biomedical Laboratories, Piscataway, NJ).

### Indirect immunofluorescence analyses (IFA)

Sub-confluent cell monolayers prepared in 12 mm glass coverslips were infected with the different FMDV strains at a MOI = 10 for the indicated time. The cells were fixed in 4% paraformaldehyde, permeabilized with 0.5% Triton X-100^®^ (Sigma) in PBS, blocked with blocking buffer (PBS, 2% bovine serum albumin [BSA], 5% normal goat serum, 10 mM glycine) and then incubated overnight at 4°C with the respective primary antibodies. FMDV VP1 was detected with mouse mAb 6HC4 (33), L^pro^ with a rabbit polyclonal Ab elicited against bacterially expressed recombinant protein (de 20), and NF-κB -p65/RelA- with rabbit polyclonal Ab-1 RB-1638 (NeoMarkers, Lab Vision). Alexa Fluor 488 and Alexa Fluor 594 (Molecular Probes, Invitrogen) conjugated secondary Abs were used for detection. Nuclei were visualized by DAPI staining included in ProLong Gold Antifade mounting media (Invitrogen). Cells were examined in an Olympus BX40 fluorescence microscope and the images were taken with a DP-70 digital camera and DP-BSW v2.2 software (Olympus America, Central Valley, PA).

### Animal experiment

Animal experiments were performed in the high-containment facility of the Plum Island Animal Disease Center following a protocol approved by the Institutional Animal Use and Care Committee (Protocol No: 151-13R). Nine Yorkshire barrows (5 weeks old, castrated males and weighing approximately 40 lbs each) were divided in three groups of three animals each. Animals were inoculated intradermally (ID) in the heel bulb of the right rear foot with FMDV A12-WT (5x10^5^ pfu/animal) [group 3, control group] or two different doses of A12-L_H138L_ (1x10^6^ or 1x10^7^ pfu/animal) [group 1 and 2, respectively] (Table 1). Rectal temperatures and clinical signs, including lameness and vesicular lesions, were monitored daily during the 1st week post-inoculation and samples of whole blood, serum and nasal swabs (BD universal viral transport [UVT] system, BD, Franklin Lakes, NJ) were collected on a daily basis to monitor complete blood counts (CBC), *in vivo* viral replication and spreading. Also, serum samples were collected at days 4, 7, 14, and 21 post-inoculation (dpi) to evaluate the development of neutralizing antibodies. Clinical scores were determined by counting the number of FMD-vesicles in the toes (max 4 vesicles per foot; 16 per animal), plus one, for one or more lesions detected in the snout and/or mouth. The maximum possible score was 17, and lesions restricted to the site of challenge were not counted (34). CBC data was done using a Hemavet^®^ analyzer (Herba Diagnostics Miami Lakes, FL), to evaluate lymphopenia.

**Table 1:** Clinical performance of swine inoculated with varying doses of FMDV A12-L_H138L_ or A12-WT.

**Group# and Dose^a^**	**Clinical results after mutant or WT virus inoculation in swine**								
	**Pig ^#^**	**Clinical Score^b^**	**Fever^c^**	**Lymphopenia^d^**	**Viremia^e^**	**Viremia-PCR^f^**	**Shedding Virus^g^**	**Shedding RT-PCR^h^**	**SN^i^**
1: A12-L_H138L_ 1 × 10^6^	29,044	3/9	Y	Y	0 / 0 / 0	Y	2 / 5.3 × 10^2^ / 3	Y	0/2.7
	29,045	0/0	N	N	0 / 0 / 0	N	3 / 1.5 × 10^1^ / 2	Y	0/3.3
	29,046	0/0	N	Y	0 / 0 / 0	N	3 / 1.5 × 10^1^ / 2	Y	0/3.0
2: A12-L_H138L_ 1 × 10^7^	29,047	3/5	N	Y	3 / 5.0 × 10^1^ / 1	Y	1 / 5.5 × 10^1^ / 1	Y	0/2.7
	29,048	3/3	Y	Y	0 / 0 / 0	Y	2 / 9.3 × 10^1^ / 2	Y	0/3.3
	29,049	2/12	Y	Y	2 / 5.0 × 10^3^ / 1	Y	2 / 4.2 × 10^2^ / 2	Y	0/3.3
3: A12-WT 5 × 10^5^	29,050	2/14	Y	Y	2 / 7.8 × 10^4^ / 2	Y	1 / 7.8 × 10^1^ / 1	Y	0/3.3
	29,051	2/7	Y	Y	2 / 7.5 × 10^1^ / 1	Y	3 / 1.5 × 10^1^ / 2	Y	0/3.3
	29,052	2/15	Y	Y	2 / 7.5 × 10^2^ / 2	Y	3 / 1.5 × 10^1^ / 1	Y	0/3.3

a Dose of inoculum per animal expressed as number of pfu in a total volume of 0.4 ml.

b Days post inoculation (dpi) first signs of lesions are detected /highest lesion score achieved throughout the entire experiment.

c Rectal temperature of ≥40°C at any time after inoculation.

d Reduced percentage of lymphocyte to ≤ 30 % of lymphocytes/mL of blood.

e First dpi that viremia was detected using virus isolation techniques; maximum amount of viremia in pfu/ml detected in sera samples; and the duration (days) of viremia.

f Any analyzed serum sample during 1 to 7 dpi was positive using Real Time-PCR (Y, yes or N, no).

g First dpi that shedding virus was detected using virus isolation techniques; maximum amount of shedding virus in pfu/ml detected in nasal swab samples; and the duration (days) of shedding.

h Any analyzed nasal swab sample during 1 to 7 dpi was positive using Real Time-PCR (Y, yes or N, no).

i SN=serum neutralizing antibody response reported as Log10 TCID50 at 0 and 21 dpi, respectively.

# Number.

### Virus titration in sera and nasal swabs

Sera and nasal swabs were assayed for the presence of virus by plaque titration on BHK-21 cells. Briefly, serial 10-fold dilutions of the samples were allowed to adsorb on monolayers of BHK-21 cells grown in 6-well plates. After 1 h adsorption, overlay was added, and the plates were incubated for 48 h at 37°C in a humidified atmosphere containing 5% CO_2_ and then stained with a crystal violet-formalin solution to visualize the plaques. Virus titers were expressed as log_10_ plaque forming units (pfu) per ml of serum or nasal swab media. Detection level ≥5 pfu/ml of serum or nasal swab media.

### Determination of neutralizing antibody titer

Neutralizing antibody titers were determined in mice or swine sera samples by end-point titration according to the Spearman-Kärber method (35). Antibody titers were expressed as the log10 value of the reciprocal of the dilution that neutralized 100 TCID_50_ in 50% of the wells.

### Analysis of cytokines in serum

IFN-α, IL-1β, IL-10 and TNF-α protein concentration was determined in sera from infected animals using an ELISA. IFN-α was detected as described above (Section 2.5). IL-10 Cytoset ELISA (Biosource-Invitrogen, Carlsbad, CA) and IL-1β, IL-6 and TNF-α Duo Set ELISAs (R&D Systems, Minneapolis, MN) were performed following the manufacturer's directions. All ELISAs were developed with 3, 3', 5, 5', tetramethylbenzidine (TMB) from KPL (Gaithersburg, MD). The absorbance at 450 nm was measured in an ELISA reader (Varioskan Lux, Thermo Fisher Scientific). Cytokine concentrations were calculated based on the optical densities obtained with the standards.

### Statistical analyses

Data handling, analysis and graphic representation were performed using Prism 5.0 (GraphPad Software, San Diego, CA) or Microsoft Excel. Statistical differences were determined using a Student's *t*, comparing the same parameter in the different groups or change of the parameter over different timepoints as compared to a baseline; Gehan-Breslow-Wilcoxon test was used to analyze clinical disease onset. Statistically significant differences were expressed with asterisks (*P* < 0.05[^*^]).

## Results

### Mutation of A12 L^pro^ H138 affects normal FMDV growth in cell culture

**Figure 1:**
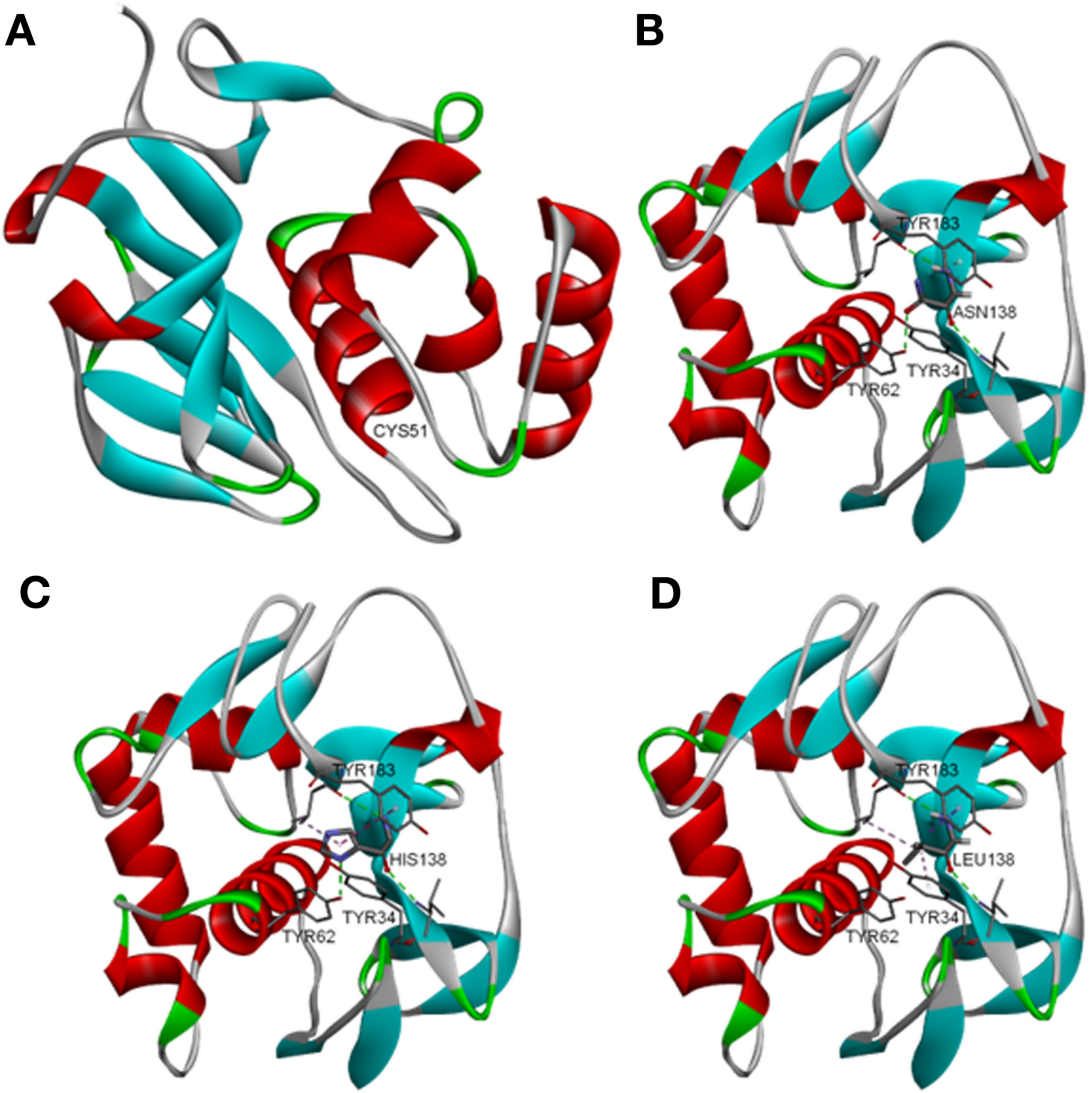
Molecular modeling of L^pro^. Structural analysis of L^pro^ reveals an aromatic pocket located opposite from the substrate binding domain. **(A)** This pocket consists of three tyrosines coordinated by an internal histidine residue (H138), which is absolutely conserved across serotypes. Simulations were performed to assess the effects of different point mutations replacing the histidine at position 138. **(B–D)** Incorporation of different mutations selectively preserved or disrupted different components of the interaction. Simulations were used to generate a collection of mutants with varying degrees of functional conservation.

**Figure 2:**
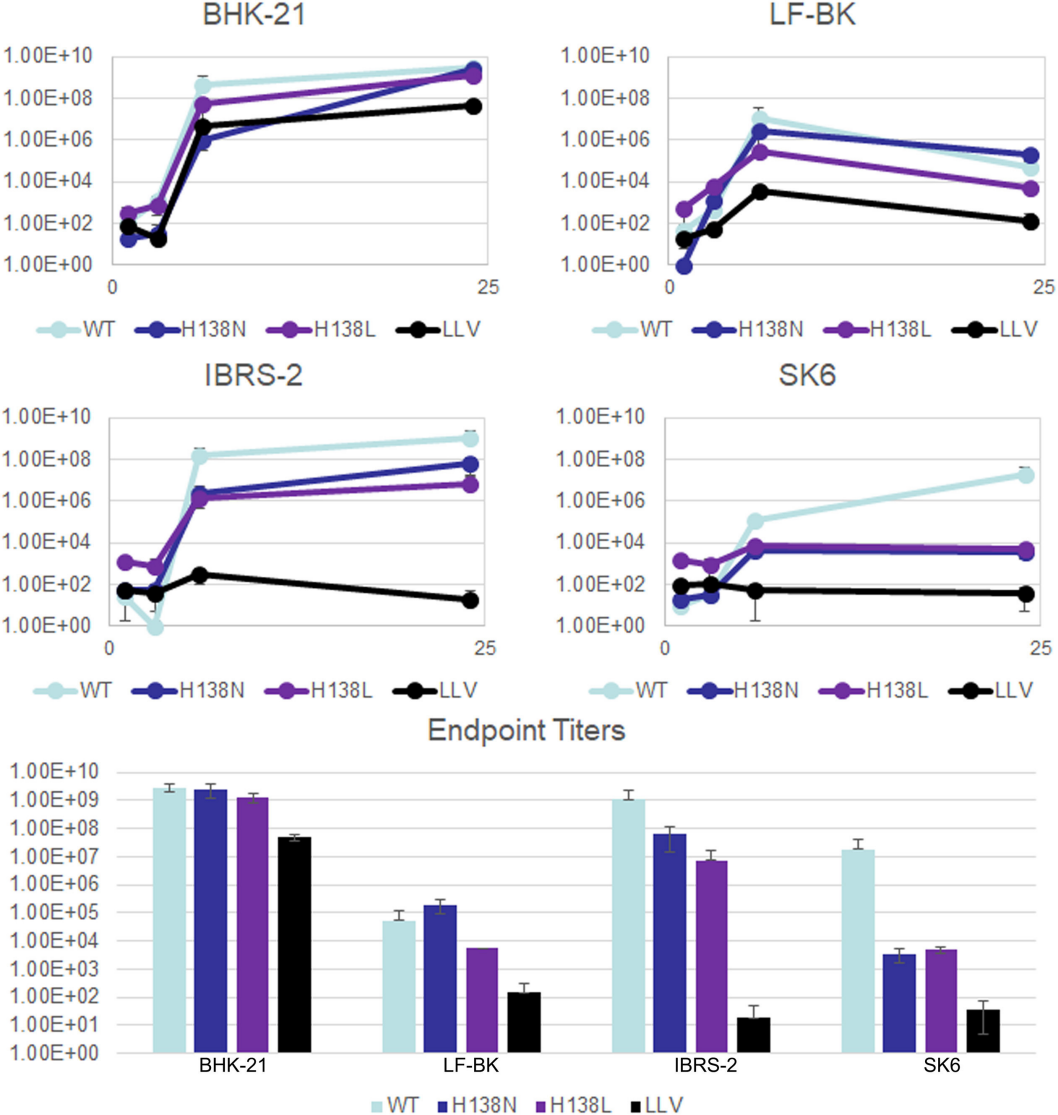
Growth kinetics for A12-L_H138_ mutants. Growth curves on BHK-21, LF-BK, IBRS2, or SK6 cells. Cells were infected with FDMV A12 wild type (WT), FMDV A12 leaderless (LLV), and FMDV A12-L_H138_ mutants (H138 N and H138 L) at MOI =10. After 1h, unabsorbed virus was removed by washing with 150 mM NaCl, 20 mM MES pH = 5.5 followed by addition of complete media. Samples were taken at 1, 3, 6 and 24 hpi and virus titers were determined by log10 TCID50 on BHK-21 cells.

A detailed structural analysis of L^pro^ revealed the presence of an aromatic pocket outside of the substrate binding domain which consists of three Tyr (Y) residues coordinated by an internal His (H138), a residue that is absolutely conserved across all FMDV serotypes (36) (Figure 1). Simulations were performed using Discovery Studio visualizer to predict the effect of different point mutations replacing residue H138 and the effect on perturbation of the aromatic pocket (leucine and asparagine shown as an example), showing that incorporation of the different mutations introduced selectively, preserved or disrupted different components of the interaction. A collection of mutants changing this specific residue was selected including H138W, H138N, and H138L, being H138W the least disruptive. By using reverse genetics, these mutations were introduced in the infectious clone but only H138N and H138L rendered viable viruses after transfection of RNA in BHK-21 cells. Viable FMDV A12-L_H138N_ and A12-L_H128L_ were used for further characterization and compared to FMDV A12-wild type (WT) and A12-Leaderless (LLV). Kinetics of growth in multiple cells lines, including BHK-21, LF-BK, IBRS-2 and SK6 cells, demonstrated that mutants of H138 have an altered growth phenotype when compared to WT virus (Figure 2). Overall, the rate of growth for H138 mutants fell between WT virus and LLV mutant virus. Also, as observed in Figure 2, A12-L_H138L_ mutant showed a lower endpoint titer specifically in LF-BK cells when compared to A12-L_H138N_. Based on these results, this mutant was further selected for additional characterization.

### Cleavage of translation initiation factor eIF-4G is only slightly affected by mutation of A12 L^pro^ H138 residue

**Figure 3:**
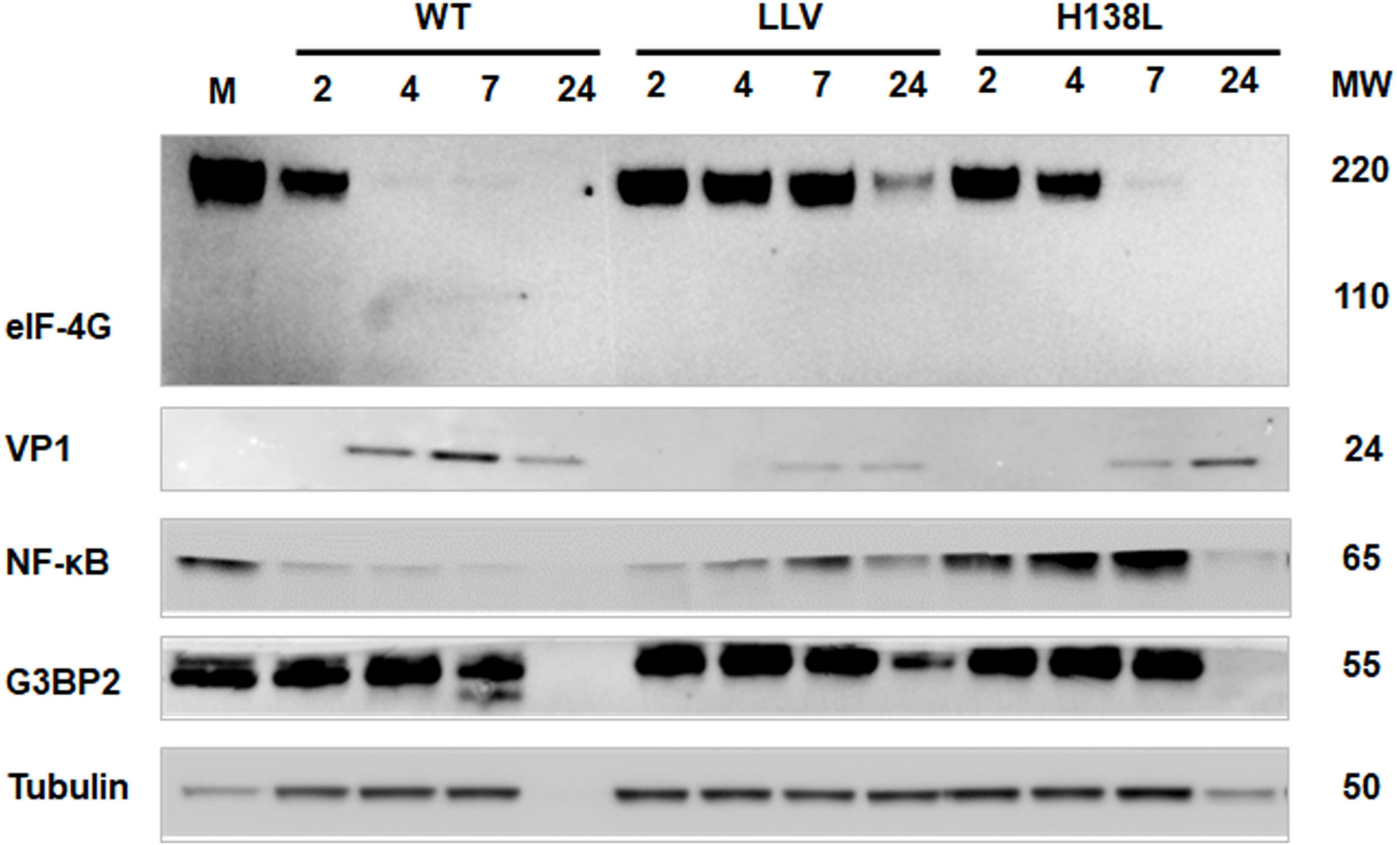
Processing of cellular proteins. LF-BK cells were infected with FMDV A12-WT, A12-LLV (leaderless) or FMDV A12-L_H138L_ at MOI = 10. Protein cytoplasmic extracts were collected at 2, 4, 7, and 24 h post infection, followed by SDS PAGE and Western Blot analyses using anti-eIF4G (p220), anti-FMDV VP1, anti-NF-κB (p65/RelA), anti G3BP2, and anti-tubulin-α antibodies. M stands for mock infected cells.

One of the hallmarks of picornavirus infection is the cleavage of the host translation initiation factor eIF-4G, an event that results in the shut-off of host cap-dependent mRNA translation (37–39). In order to determine if mutations in H138 residue affected eIF-4G cleavage, we performed western blot analysis on protein extracts obtained from LF-BK cells infected with FMDV A12-L_H138L_, in comparison to FMDV A12-WT. We also run a parallel infection with FMDV A12-LLV which is known as unable to process eIF-4G (13). As shown in Figure 3, by 4 h post infection, cellular factor eIF-4G (p220) was almost completely processed by A12-WT virus. Interestingly only a slight delay, ~2 h, was observed in cells infected with A12-L_H138L_ although cleavage was complete thereafter. As expected, little or no processing was observed in the time lapsed when A12-LLV was used. This result suggested that alterations of viral growth of A12-L^pro^H138L were probably not due to the overall shut-off of cellular translation imposed by L^pro^.

### NF-κB (p65/RelA) and G3BP2 are not cleaved upon infection with FMDV A12-L_H138L_

We have previously demonstrated that during FMDV infection there is L^pro^ dependent degradation of NF-κB that correlated with nuclear accumulation of L^pro^ (20). Further, mutations in L^pro^ SAP (**S**AF-A/B, **A**cinus and **P**IAS) domain had an effect in L^pro^ nuclear retention and degradation of NF-κB. Analysis of protein profiles in lysates of infected cells indicated that mutation H138L in L^pro^ directly affects degradation of NF-κB. As seen in Figure 3, by 4 hpi, p65/RelA signal was significantly reduced in the cytoplasm of LF-BK cells infected with A12-WT. In contrast, the p65/RelA signal did not decrease in the extracts of A12-L_H138L_ infected cells, and signal resembled that of mock infected cells (Figure 3). Parallel analysis of NF-κB by indirect immunofluorescence analysis to evaluate the localization of p65/RelA in infected cells demonstrated that by 4 hpi, the p65/RelA signal was almost absent from the nucleus of A12-WT infected cells while it accumulated in the nucleus of A12-LLV infected cells (Supplementary Figure 1), as previously reported (20). Interestingly, the pattern for A12-L_H138L_ infected cells resembled the pattern for A12-LLV, with a bright p65/RelA staining concentrated in the nuclei (Supplementary Figure 1). The same image was observed even after 8 hpi (data not shown), time point considered a relatively late stage of infection in LF-BK cells which are known to be very susceptible to FMDV infection (40, 41).

It has also been reported that FMDV antagonizes the innate immune response, by modulating the stress response (24). In particular, it has been known that L^pro^ causes degradation of the scaffold proteins Ras GTPase-activating protein-binding proteins 1 and 2 (G3BP1 and G3BP2), preventing stress granules formation and accumulation (24). Interestingly, no cleavage of G3BP2 was detected in FMDV A12-L_H138L_ infected cells (Figure 3).

### Mutation in A12 L^pro^ H138 residue does not affect L^pro^ nuclear retention

**Figure 4:**
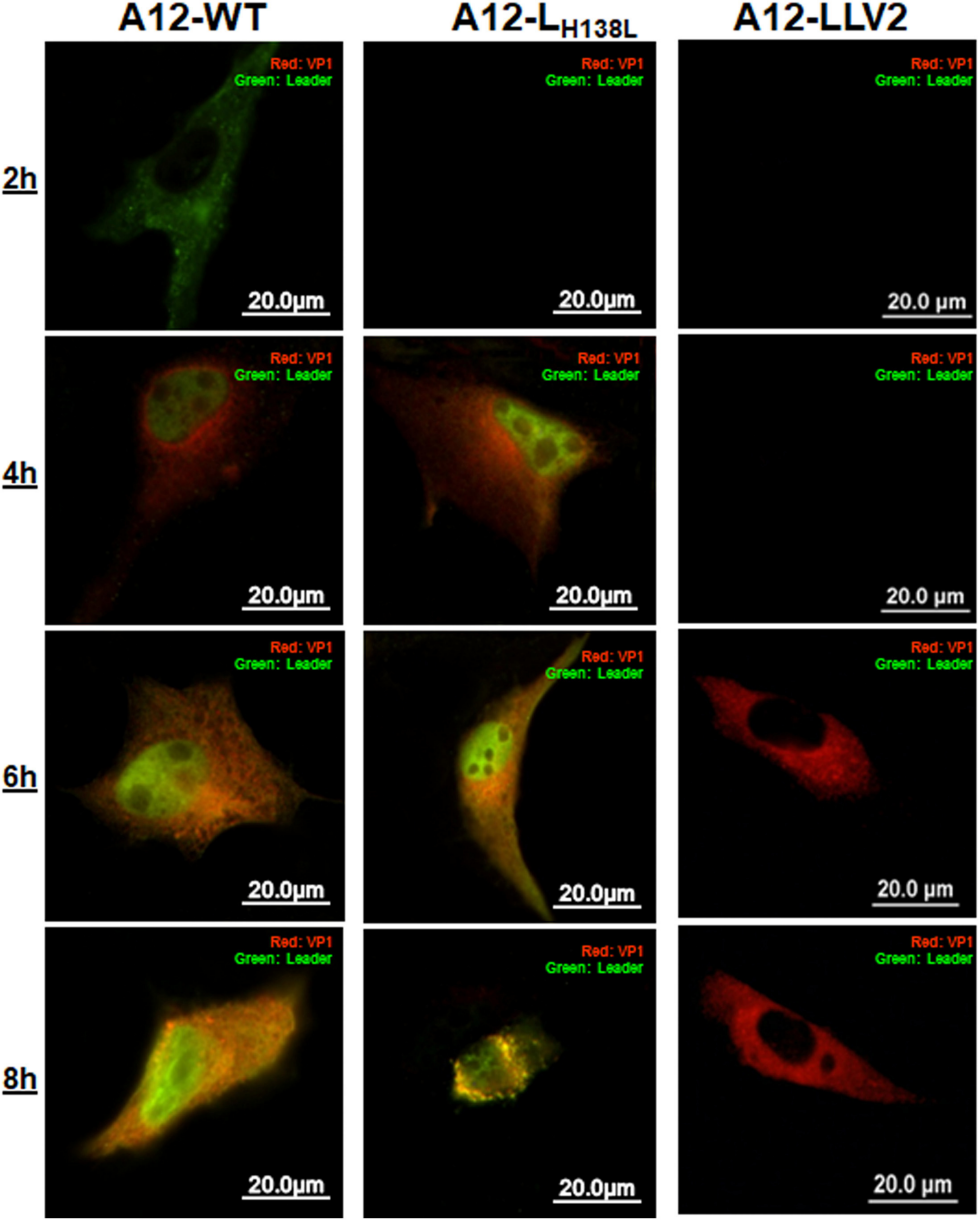
Translocation of L^pro^ is not affected in A12-L_H138L._ LF-BK cells were infected with A12-WT, A12-L_H138L_ or A12-LLV FMDV at MOI = 10. At the indicated times cells were fixed and viral proteins L^pro^ (green) and VP1 (red) were detected by IFA.

Previous results reported by our lab suggested that nuclear accumulation of L^pro^ is required for p65/RelA degradation, since during infection with L^pro^ SAP mutant, no L^pro^ could be detected in cell nuclei at relatively late times post infection while little or no NF-κB degradation could be detected in the same compartment (26). We analyzed the sub-cellular localization of L^pro^ during infection with A12-WT, A12-LLV2, and A12-L_H138L_. As observed in Figure 4, cells infected with A12-WT showed an early signal (2 hpi) of L^pro^, that rapidly appeared and accumulated in cell nuclei. A similar pattern was observed after infection with A12-L_H138L_, although detection of mutant L^pro^ was slightly delayed in comparison to WT L^pro^. As expected, no L^pro^ was detected in cells infected with leaderless A12-LLV virus.

In sum, the results of this study indicated that disruption of the L^pro^ H138 residue did not affect the ability of L^pro^ to translocate to the nucleus of infected cells or to target the general translation factor eIF-4G, but selectively prevented, at least, p65/RelA and G3BP2 proteolytic processing.

### Mutation of A12-L^pro^ H138 residue prevents L^pro^ inhibition of IFN expression

**Figure 5:**
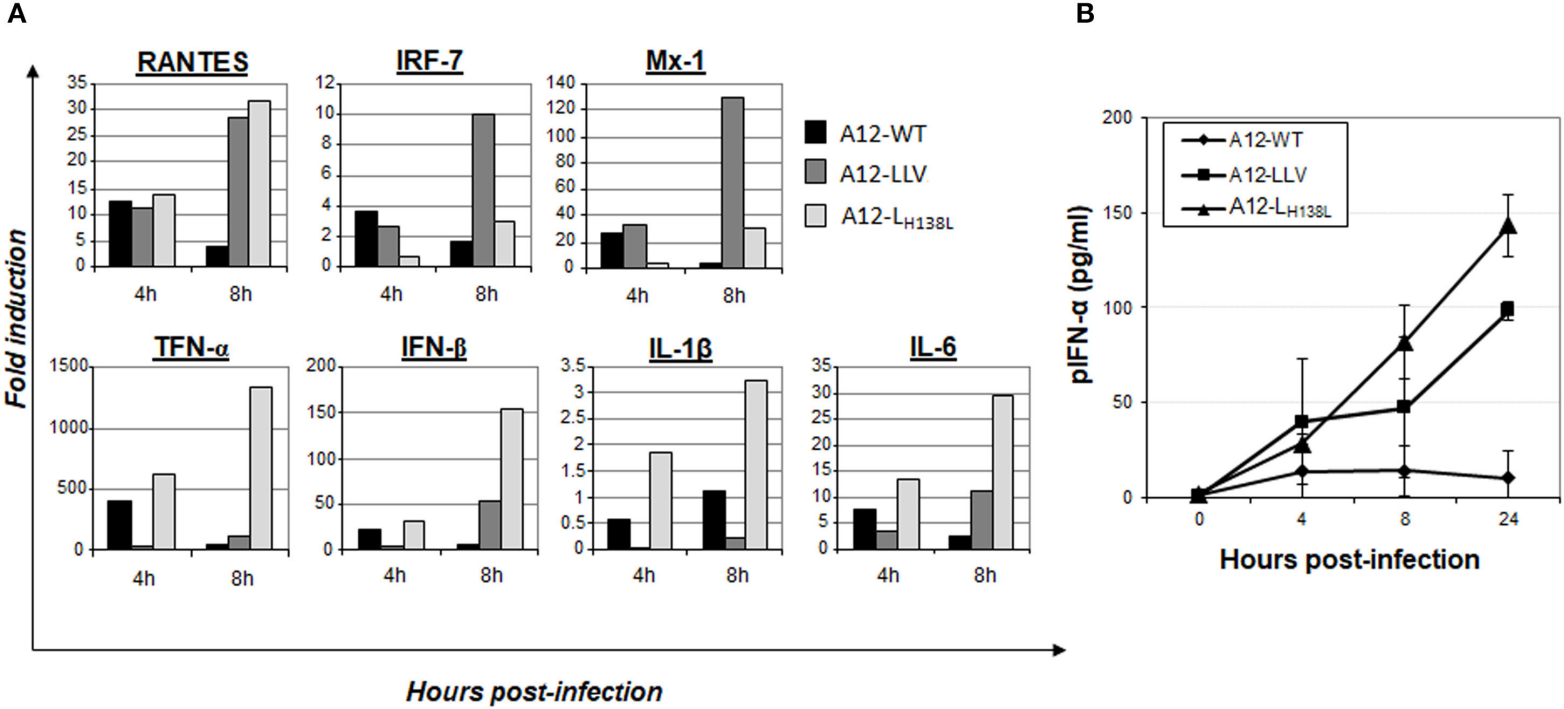
Induction of IFN and ISGs during infection. **(A)** Expression of IFN-β, TNF-α, RANTES, Mx1, IL-1β and IL-6 and IRF7 mRNAs was measured by real-time RT-PCR in primary porcine kidney cells infected with A12-WT, A12-LLV and A12-L_H138L_ at MOI = 10. Graphs represent results of one out three independent studies with parallel results. **(B)** Porcine IFNα ELISA in supernatants of PK cells infected with A12-WT, A12-LLV and A12-L_H138L_, plotted as AVER±. STDEV of three independent studies.

It is well-stablished that L^pro^ antagonizes the innate immune response by blocking the expression of IFN and NF-κB signaling (23, 25, 42). To test whether mutation of L^pro^ H138 residue affects the FMDV capability of blocking IFN expression, we analyzed the levels mRNA for IFN-β, the pro-inflammatory cytokine TNF-α, the chemokine RANTES and the IFN stimulated genes (ISGs) Mx1 and IRF7. For this analysis we used cells known to have intact IFN responses such as primary/secondary porcine kidney (PK). For comparison we also infected cells with A12-WT and A12-LLV2 (Figure 5). Although by 4 hpi the differences among expression varied among the analyzed genes a clear pattern of upregulation was seen in all analyzed transcripts at 8 hpi, with significantly higher expression in cells infected with A12-L_H138L_ and A12-LLV than in cells infected with A12-WT (Figure 5A). Interestingly, most of the analyzed genes, except for IRF-7 and Mx1, showed higher expression upon infection with A12-L_H138L_ as compared to A12-LLV. ELISA quantitation of secreted IFNα protein in the supernatants of infected PK cells, followed similar kinetics (Figure 5B). By 24 hpi, 5 to 8-fold higher amounts of IFN-α protein were detected in A12-LLV and A12-L_H138L_, respectively, as compared to A12-WT. These results indicated that mutation on A12-L^pro^ H138 residue prevented the inhibitory effect of L^pro^ on NF-κB dependent transcriptional activity and IFN protein expression.

### FMDV A12-L_H138L_ mutant is attenuated in swine

To compare virulence of A12-L_H138L_ vs. A12-WT *in vivo*, we inoculated animals intradermally (ID) in the rear heel bulb (*n* = 3) with either 10^5^ pfu/animal of A24-WT (group 3/control group) or two different doses of A12-L_H138L_ (group 1: 10^6^ pfu/animal and group 2: 10^7^ pfu/animal) (Table 1). A12-WT inoculated animals showed vesicles as early as 2 days post-inoculation (dpi) (Table 1) and transient fever and lymphopenia were also detected on the day of disease onset or 1 day later, as previously described (43). However, when animals were inoculated with a 10-fold higher concentration of the A12-L_H138L_ virus, only one out of three animals showed vesicles, lymphopenia and fever with 1 day delayed disease onset. Furthermore, even when the animals were inoculated with a 100-fold higher dose of A12-L_H138L_ than WT virus, disease onset was statistically significantly delayed on those animals as compared to animals inoculated with A24-WT virus (*P*< *0.05*) (Table 1).

Animals inoculated with A12-WT developed viremia concomitantly with the appearance of clinical signs. Interestingly, none of the animals inoculated with A12-L_H138L_ at 1x10^6^ pfu had detectable viremia measured either by virus isolation or by rRT-PCR, and one out of three animals inoculated with 1 × 10^7^ pfu of A12-L_H138L_ did not show any viremia (Table 1). On the other hand, virus shedding was detected in nasal swabs in all inoculated animals regardless the virus or dose used (Table 1).

All together, these data indicate that A12-L_H138L_ FMDV displays significantly reduced virulence in swine as compared to A12-WT.

### FMDV A12-L_H138L_ mutant and A12-WT elicit equivalent adaptive immune responses

It has previously been demonstrated that animals inoculated with an attenuated strain of FMDV with mutations or complete deletion of L^pro^, developed significant increase of antibody titers against viral proteins in swine serum (15, 27). In the current experiment, we observed that regardless the presence or absence of viremia, all the animals inoculated with A12-L_H138L_ developed significant levels of FMDV-specific neutralizing antibodies starting at 7 dpi with a peak at 14 dpi (Table 1), and there was not statistically significant difference in the virus titers between A12-WT or any of the A12-L_H138L_ inoculated groups (*P* > *0.05*).

### Mutations in A12-L^pro^ H138 residue has an effect on cytokine profile in swine

**Figure 6:**
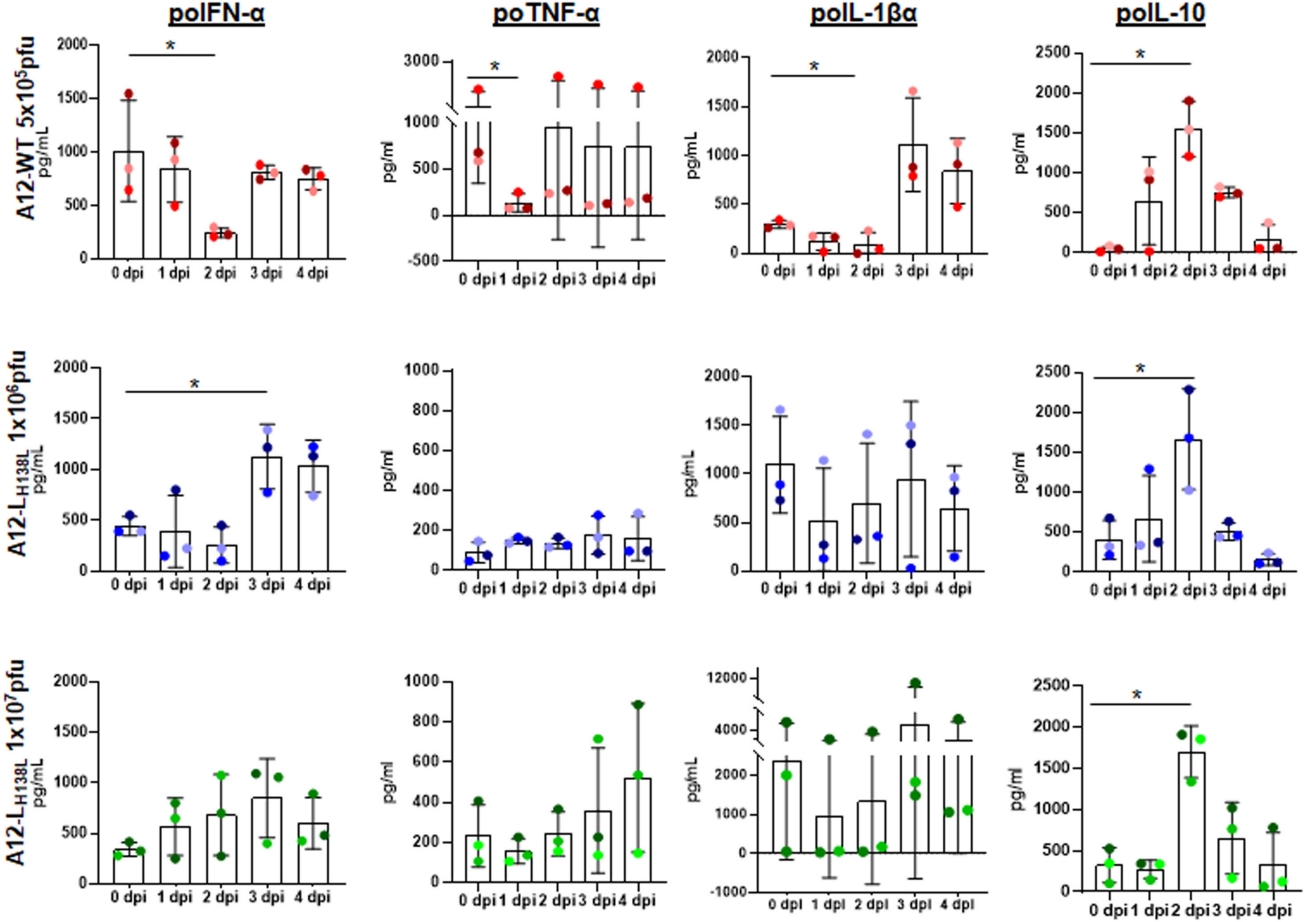
Cytokine profile in animals inoculated with FMDV A12-WT or A12-L_H138L_. Serum levels of porcine IFN-α, IL-10, IL-1β, and TNF-α were detected by ELISA at different times post virus inoculation. Results are expressed in pg/ml. Error bars represent the variation within the three animals from each group. Each color grade dot represents the same animal throughout the graphs (*P* < 0.05[*]).

We have previously demonstrated that L^pro^, is an immune response antagonistic factor, limiting the expression of IFN and ISGs (17–21), and reducing the expression of pro-inflammatory cytokines IL-1β, IL-6 and TNF-α in swine (27). Furthermore, FMDV WT infection induces production of the anti-inflammatory cytokine IL-10, thus impairing T-cell proliferation (44) with a consequent induction of an anti-inflammatory state. To understand if H138 residue is involved in this effect, we analyzed the expression of pro- and anti-inflammatory cytokine protein levels in the sera of animals inoculated with A12-L_H138L_ mutant and A12-WT for 5 days after infection. In the case of A12-WT inoculated animals, despite a high variability among individuals, a statistically significant decrease was observed of all analyzed pro-inflammatory cytokines (IFN-α, TNF-α, and IL1-β) between days 1 and 2 post-inoculation (Figure 6). Furthermore, there was an increase in the levels of IL-10 with a peak at 3 dpi (Figure 6). Similarly, animals inoculated with A12-L_H138L_ showed a peak of IL-10 at 3 dpi, regardless of the dose of virus inoculated. However, when analyzing the expression of pro-inflammatory cytokines, a different profile was detected. None of the animals inoculated with A12-L_H138L_ showed a decrease in any of the pro-inflammatory cytokines analyzed. On the contrary, in the case of IFN-α, animals inoculated with 1 × 10^6^ pfu of A12-L_H138L_ showed a consistent increase starting at day 3 post-inoculation (Figure 6). These results indicate that mutations in L^pro^ residue H138 alter the capacity of FMDV to counteract the *in vivo* pro-inflammatory immune response.

## Discussion

As all viruses, FMDV has co-evolved with its host to successfully antagonize the immune response. In the particular case of innate responses, despite one protein, L^pro^ stands out as the most effective IFN suppressor (23, 42, 45, 46). As Vadim Agol described several years ago, FMDV L^pro^, and most picornaviral 2A proteins, could be grouped as “security proteins, dedicated specifically to counteract host defenses,” bearing non-essential activities on the viral polyproteins (47). Consistently, deletion of FMDV L^pro^ is tolerated and results in a viable virus that can grow well in immortalized cells with no selective immune pressure (13); however, infectiousness in the animal host is severely affected (14, 15). In contrast, mutations that target the catalytic pocket of the enzyme result inactive protein and non viable viruses (13, 38). Interestingly, mutations that lie outside the L^pro^ catalytic pocket and at the 3' end of the protein are usually tolerated (3, 22, 26, 27, 48).

Here we demonstrate as a proof-of-concept that another L^pro^ mutation that resides outside the catalytic site, H138L, is tolerated by FMDV, resulting in a mutant virus with an attenuated phenotype *in vitro* and *in vivo*, in swine. This mutation lies in a His residue that seems to stabilize an aromatic pocket of the protein containing three Tyr residues and that is highly conserved across all FMDV serotypes (36). Indeed, earlier work by Piccone et al. had reported that viruses containing this mutation were not attenuated in cell culture displaying a WT virus phenotype (13). However, the authors had only used immortalized cells (BHK-21) that lack features of a natural host (49, 50).

We also observed that in SK6 cells, an immortalized cell line with an apparently normal IFN pathway, A12-L_H138L_ mutant is significantly more attenuated than in cell lines that display an altered IFN induction or transduction pathways (18). Consistent with this observation, L^pro^ dependent degradation of innate immunity molecules such as p65 (NF-κB), or stress granules marker G3BP2, was less evident in cells infected with A12-L_H138L_ mutant, while only a mild delay was observed in cleavage and degradation of the translation initiation factor eIF-4G. As a result, higher levels of IFN and ISGs were detected in cells infected with A12-L_H138L_ as compared to WT virus (Figure 3). These results resembled previous observations made with the FMDV mutant L^pro^W105A that mostly affected the deISGylation capability of L^pro^ without affecting the specific cleavage of translation factors (22, 51). Analysis of deUbiquitinase activity indicated that similarly to LLV, A12-L_H138L_ had a reduced ability to decrease the ubiquitination profile of host proteins (data not shown). Perhaps reduced stability or misfolding of A12-L_H138L_ protein partially affects ubiquitin dependent signaling in response to viral infection. Further research including detailed biochemical studies is warranted to confirm this hypothesis. Similar results have been observed for other RNA viruses such as Middle East respiratory syndrome coronavirus (MERS-CoV), severe acute respiratory syndrome coronavirus (SARS-CoV-1 and SARS-CoV-2), mouse hepatitis virus (MHV), and porcine reproductive and respiratory syndrome virus (PRRSV) (52–54), emphasizing the role of posttranslational modification in intrinsic protein function or interaction with other factors.

Overall, our results confirmed that intact/unmutated L^pro^ is required for an effective suppression of the cellular innate response against FMDV infection. While only a slight delay was detected in the processing of eIF-4G, a significant increase in the expression of IFN and ISG was detected in cells infected with A12-L_H138L_ as compared to WT. However, differently than for other L^pro^ mutations, the mutation H138L did not affect the ability of the protein to accumulate in the nucleus of infected cells and interestingly, no p65 degradation could be detected, despite the normal processing of eIF-4G. Similar results had previously been shown for L^pro^ SAP mutant, but it is worthy to mention that this mutant did not accumulate in the nucleus of infected cells, suggesting that this behavior might have affected p65 degradation (26). Interestingly, another L^pro^ mutant (L^pro^ L143A) known to affect substrate specificity at least *in vitro*, (39) did not induce degradation of p65 or cleavage of RLR signaling proteins (i.e., MAVS, TBK), but preserved the induction of high levels of IFN-β mRNA transcripts (51). Hence, mutation in H138 may interfere or prevent the interaction with p65 or other protein of a multiprotein complex thus far unknown. In parallel, A12-L_H138L_ mutant failed to cause cleavage/degradation of the stress granule component G3BP2. Analysis of ISG mRNA and IFN protein expression revealed that this mutant behaved similarly to FMDV LLV. Both, LLV and A12-L_H138_ mutants induced higher levels of ISGs and IFN than the WT virus. These results are consistent with our previous experiments using LLV and the SAP L^pro^ mutants (20, 26) but different than those obtained for A12-L^pro^W105A or other nearby mutants in which the deUb/deISG activity was significantly impaired while ISG expression was not blocked (22, 51). Although induction of innate immune responses based on the examination of some mRNA transcripts (Mx1 and IRF7) was better induced by A12-LLV, overall A12-L_H138L_ produced a stronger response (higher upregulation in RANTES, TNF-α, IFN-β, IL-1β, and IL-6 transcripts). It is possible that not all IFN dependent promoters do get activated at the same time, or a higher replication rate for A12-L_H138L_ as compared to LLV must have induced the pathway more efficiently. In fact, levels of replication of A12-L_H138L_ were always higher than LLV in all cell lines used. On the other hand, the picture was different when A12-L_H138L_ and WT titers were compared. While no differences in viral titers were detected in cell lines with impaired IFN signaling systems, the titers of A12-L_H138L_ were significantly lower than those for WT in cells which display a competent IFN system (49, 50) such as SK6 cells.

These results may also explain the phenotype observed in the animal experiments in which two out of three pigs inoculated with twice the dose of A12-L_H138L_ virus as compared to WT virus, did not develop clinical signs of FMD, neither they had viremia. Further, two pigs inoculated with 20 times higher dose also showed less severe disease and in one of them no virus could be isolated from serum. Consistent with these results, animals inoculated with the mutant virus that did not get sick or had lower scores in the evaluation of clinical signs and developed higher levels of systemic IFN protein. These results were also similar to those previously obtained with the SAP FMDV mutant (27). The levels of systemic IFN significantly increased early after inoculation of swine with FMDV A12-L_H138L_, while they decreased or remained unchanged in animals inoculated with WT virus. In contrast, the levels of proinflammatory cytokines (TNF-α and IL-1β) were not affected in animals infected with the mutant virus while they significantly decreased in animals inoculated with WT virus prior to the appearance of clinical signs. It has recently been proposed that the IL-1 family of cytokines may act as an important backup antiviral system in the host with a critical role in skin defense (55). Production of IL-1 by keratinocytes induces an antiviral state in neighboring stromal cells including among others, fibroblasts and endothelial cells. Of interest, this effect was seen in tissues infected with fully virulent strains of VSV and Zika virus. It is possible then that FMDV has also evolved to block this response in the host, causing significant damage in skin tissues, and L^pro^ may contribute a pivotal role for the pathogenesis of the virus.

Similarly, it has been shown that TNF promotes a dual function: it provides protective antiviral immunity; and, at the same time, it enhances inflammation (56). For example, poxviruses and herpesviruses A, produce soluble or secreted versions of TNF like receptors that could neutralize this host cytokine. Also, regulation of IFN-β and TNF-α have previously been associated with acute and persistent phases of FMDV infection (57, 58). As seen in our experiments *in vitro*, it is plausible to think that higher expression of IL-1 and TNF-α are the result of the reduced degradation of NF-κB for the FMDV A12-L_H138L_ mutant as compared to the WT virus, however further pathogenesis studies are required to confirm this hypothesis. Though, similar increases in the levels of the cytokine IL-10 were detected for both, FMDV A12-L_H138L_ and WT virus. The role of IL-10 in modulation of dendritic cell (DC) function early post infection, has been studied (59). Presumably this cytokine induces a Th2 /cytokine-like environment, and as a consequence high levels of FMDV-specific neutralizing antibodies are induced (44). On this regard, similar levels of IL-10 were detected in the animals infected with WT and A12-L_H138L_ viruses consistent with equivalent levels of detectable neutralizing antibodies by 21 days post infection. Our results are consistent with previous studies with other FMDV mutants (27) and further support the concept of IL-10 as key regulatory cytokine during FMD (60).

In summary, our results further support the notion that manipulation of the L^pro^ coding region is perhaps the best tool to derive live attenuated strains of FMDV. Further studies with a larger number of testing individuals should be performed to demonstrate statistical power. A fine tune of attenuation is imperative to partially block the host innate responses while allowing for sufficient viral replication and induction of strong adaptive immune responses. A delicate manipulation of L^pro^ in the context of other genetic changes including the incorporation of DIVA markers, may help develop improved live attenuated vaccine candidates to be evaluated in different endemic settings.

## Data availability statement

The original contributions presented in the study are included in the article/Supplementary material, further inquiries can be directed to the corresponding author/s.

## Ethics statement

The animal study was reviewed and approved by Plum Island Animal Disease Center Animal Care and Use Committee (protocol #151-13R).

## Author contributions

FD-SS and TS conceived and supervised the study. PA and DR performed structural analysis, designed and/or constructed mutant viruses. PA, GM, ER-M, MR-C, ES, and FD-SS performed laboratory and/or animal experiments. JZ and ER-M provided support and critical thinking on the research design. PA, GM, FD-SS, and TS wrote the manuscript. All authors contributed to the article and approved the submitted version.

## Funding

This research was supported in part by the Plum Island Animal Disease Research Participation Program administered by the Oak Ridge Institute for Science and Education through an interagency agreement between the U.S. Department of Energy and the U.S. Department of by CRIS project number 1940-32000-061-00D, ARS, USDA, and National Pork Board Project #11-005.

## Conflict of interest

Author DR is currently employed by the company Pfizer Worldwide Research.

The remaining authors declare that the research was conducted in the absence of any commercial or financial relationships that could be construed as a potential conflict of interest.

## Publisher's note

All claims expressed in this article are solely those of the authors and do not necessarily represent those of their affiliated organizations, or those of the publisher, the editors and the reviewers. Any product that may be evaluated in this article, or claim that may be made by its manufacturer, is not guaranteed or endorsed by the publisher.

## Supplementary material

Supplementary Figure 1 Nuclear p65/RelA (NF-κB) is not degraded during A12-LH138L early infection. LF-BK cells were infected with A12-WT, A12-LH138L or A12-LLV FMDV at MOI=10. At 4h post infection cells were fixed and p65/RelA (NF-κB) and viral protein VP1 were stained and detected by IFA. Mock and poly r[IC] treated were used as negative and positive controls to verify proper p65 response/cellular localization.
