# Supplementary figures and images for: Mutation of FMDV Lpro H138 residue drives viral attenuation in cell culture and in vivo in swine
Source: Front Vet Sci. 2022 Oct 31;9:1028077. doi: 10.3389/fvets.2022.1028077 (PMC9661595; doi:10.3389/fvets.2022.1028077)

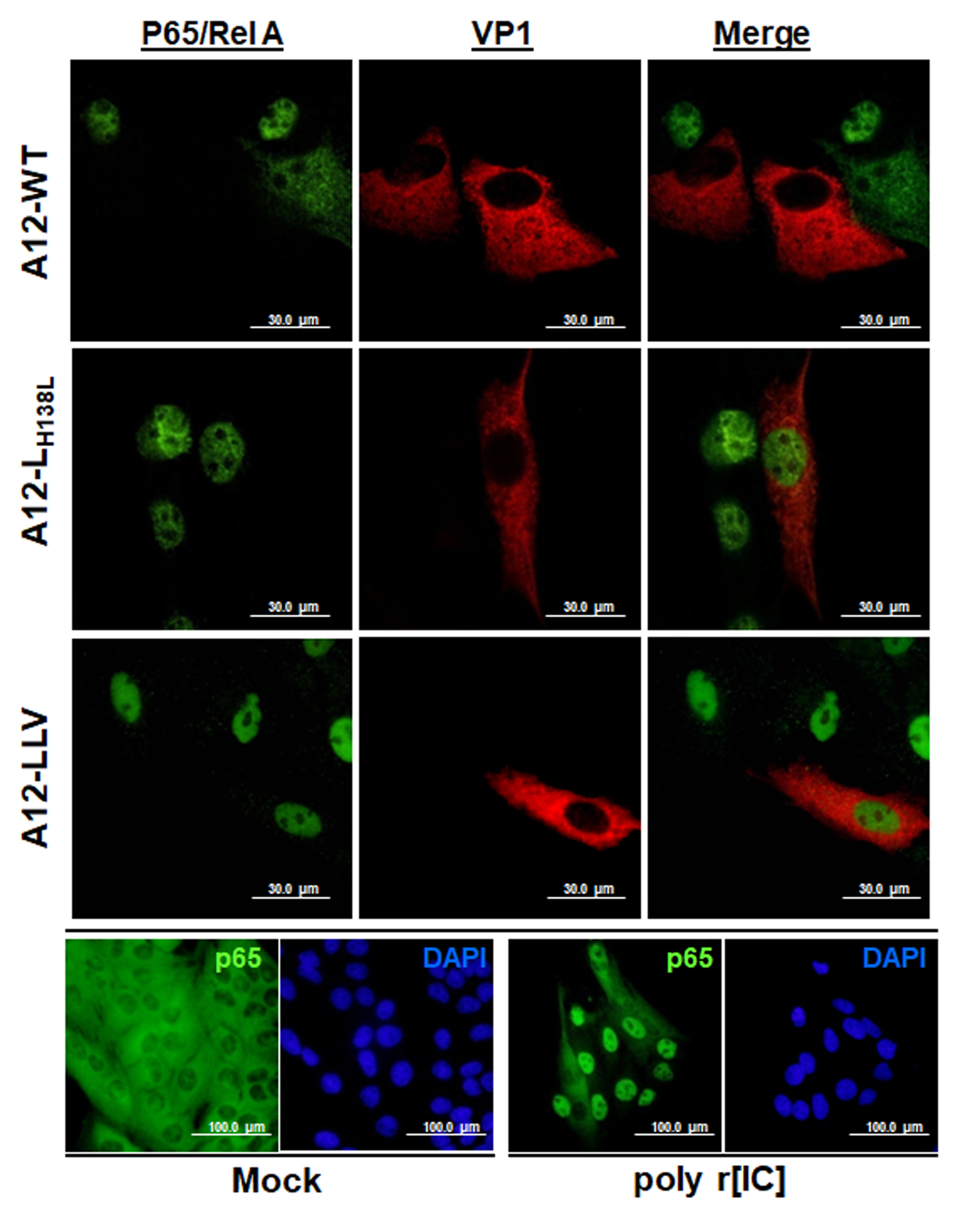

Supplement: Supplementary Figure 1 — Nuclear p65/RelA (NF-κB) is not degraded during A12-LH138L early infection. LF-BK cells were infected with A12-WT, A12-LH138L or A12-LLV FMDV at MOI=10. At 4h post infection cells were fixed and p65/RelA (NF-κB) and viral protein VP1 were stained and detected by IFA. Mock and poly r[IC] treated were used as negative and positive controls to verify proper p65 response/cellular localization. [file Image_1.TIF]
